# Brassinosteroid signaling directs formative cell divisions and protophloem differentiation in *Arabidopsis* root meristems

**DOI:** 10.1242/dev.145623

**Published:** 2017-01-15

**Authors:** Yeon Hee Kang, Alice Breda, Christian S. Hardtke

**Affiliations:** Department of Plant Molecular Biology, University of Lausanne, Biophore Building, Lausanne CH-1015, Switzerland

**Keywords:** *Arabidopsis*, Root, Brassinosteroid, Phloem, BRX, OPS

## Abstract

Brassinosteroids (BRs) trigger an intracellular signaling cascade through its receptors BR INSENSITIVE 1 (BRI1), BRI1-LIKE 1 (BRL1) and BRL3. Recent studies suggest that BR-independent inputs related to vascular differentiation, for instance root protophloem development, modulate downstream BR signaling components. Here, we report that protophloem sieve element differentiation is indeed impaired in *bri1 brl1 brl3* mutants, although this effect might not be mediated by canonical downstream BR signaling components. We also found that their small meristem size is entirely explained by reduced cell elongation, which is, however, accompanied by supernumerary formative cell divisions in the radial dimension. Thus, reduced cell expansion in conjunction with growth retardation, because of the need to accommodate supernumerary formative divisions, can account for the overall short root phenotype of BR signaling mutants. Tissue-specific re-addition of BRI1 activity partially rescued subsets of these defects through partly cell-autonomous, partly non-cell-autonomous effects. However, protophloem-specific BRI1 expression essentially rescued all major *bri1 brl1 brl3* root meristem phenotypes. Our data suggest that BR perception in the protophloem is sufficient to systemically convey BR action in the root meristem context.

## Abstract (precis)

**Highlighted article:** In addition to controlling cell expansion, brassinosteroid signaling acts cell-autonomously and non-cell-autonomously to promote protophloem differentiation and restrict formative divisions in root meristems.

## INTRODUCTION

Brassinosteroids (BRs) were discovered as plant cell elongation and division stimulants. Although the principally active BR, brassinolide, was isolated in 1979 (Grove et al., 1979), the BR signaling pathway was characterized much later, through genetic approaches in *Arabidopsis thaliana*. The key discovery was the isolation of BRASSINOSTEROID INSENSITIVE 1 (BRI1), a receptor kinase that triggers an intracellular signaling cascade upon extracellular BR perception (Li and Chory, 1997). Downstream BR signaling involves the GSK3/SHAGGY-LIKE kinase BRASSINOSTEROID-INSENSITIVE 2 (BIN2) and its substrates, the homologous transcription factors BRASSINAZOLE-RESISTANT 1 (BZR1) and BRI1-EMS-SUPPRESSOR 1 (BES1) (Li and Nam, 2002; Yin et al., 2002; Zhu et al., 2013). BR perception inactivates BIN2, thereby enabling BZR1 and BES1 to promote a transcriptional response. Among the three *Arabidopsis* BRI1 homologs, only BRI1-LIKE 1 (BRL1) and BRL3 are functional BR receptors (Cano-Delgado et al., 2004). *brl1* or *brl3* single or double loss-of-function mutants lack discernible phenotypes; however, the severe dwarfism of *bri1* mutants is enhanced in *bri1 brl1 brl3* triple mutants. Interestingly, tissue-specific expression of *BRI1* in the epidermal cell layer largely recues *bri1* dwarfism (Savaldi-Goldstein et al., 2007), but cannot complement defects in internal tissues, for example altered vascular patterning.

The role of BRs is best understood in hypocotyl elongation. Here, BR signaling is required for optimal cell expansion, which involves synergistic interaction with other hormones, for instance auxin (Nemhauser et al., 2004). BRs are also essential for primary root growth, because loss-of-function biosynthetic as well as signaling mutants have short roots. Whereas the *bri1* shoot phenotype appears to primarily result from reduced cell expansion (Savaldi-Goldstein et al., 2007), the situation is more complex in the root, where cell proliferation and elongation are deeply intertwined (Scacchi et al., 2010). In root development, BR impact on cell proliferation is seemingly more prominent (Gonzalez-Garcia et al., 2011; Hacham et al., 2011). For example, it was reported that root meristem size is reduced in *bri1* mutants as indicated by the number of dividing cells along cortex cell files. Although such reduction was not observed in a BR biosynthesis mutant (Chaiwanon and Wang, 2015), all mutants displayed decreased cell elongation, as indicated by, for example, mature cortex cell length. Phenotypic discrepancies could be explained by redundancies or cross-regulations, for instance between BR receptors, or by alternative biosynthetic bypasses (Ohnishi et al., 2006). Moreover, analysis of tissue-specific *BRI1* re-addition into receptor mutants led to the conclusion that epidermal *BRI1* activity promotes root meristem growth and affects inner tissues through unknown non-cell-autonomous signals (Hacham et al., 2011; Vragović et al., 2015).

Recently, it was found that GSK3 activity, including BIN2, could be modulated by a non-BR receptor system that controls xylem vessel differentiation (Cho et al., 2014; Kondo et al., 2014). Moreover, it was reported that BIN2 is a crucial interactor of OCTOPUS (OPS) (Anne et al., 2015), a quantitative positive master regulator of protophloem differentiation in the root meristem (Rodriguez-Villalon et al., 2014; Truernit et al., 2012). Loss-of-function *ops* mutants display impaired protophloem sieve element differentiation, which is associated with systemic effects, such as reduced auxin activity throughout the meristem and strongly reduced root growth. Loss-of-function mutants in *BREVIS RADIX* (*BRX*), another positive regulator of protophloem differentiation, have a similar phenotype (Rodriguez-Villalon et al., 2015). Interestingly, it was reported that *brx* root growth could be partially rescued by BR application (Gujas et al., 2012; Mouchel et al., 2006), and *ops* protophloem defects could be partially suppressed by BR pathway activation (Anne et al., 2015; De Rybel et al., 2009). These observations motivated us to assess the role of BR signaling in root protophloem differentiation.

## RESULTS

### BR pathway activation cannot substitute for known protophloem differentiation factors

**Fig. 1.**
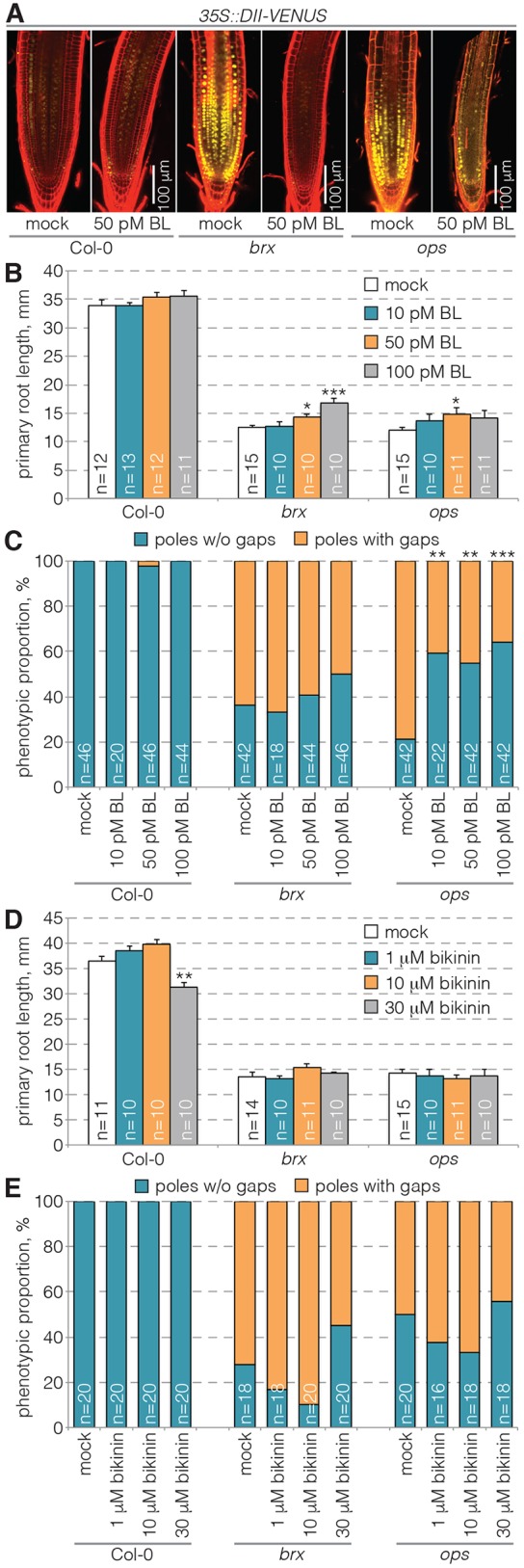
**Partial rescue of protophloem or root growth differentiation defects in *brx* and *ops* mutants by stimulation of brassinosteroid signaling.** (A) Auxin response in *Arabidopsis* root meristems upon brassinolide (BL) treatment as indicated by the inverse fluorescent DII-VENUS marker (yellow) in Col-0 wild-type, *brx* mutant or *ops* mutant background. Confocal microscopy images of propidium iodide-stained (red) meristems from 7-day-old plants that were transferred onto BL media for 3 days at 4 days old are shown compared with plants continuously grown on standard media (mock). (B-E) Quantification of root growth (B,D) and protophloem sieve element differentiation defects (‘gaps’; C,E) in seedlings transferred onto media containing the indicated molecules for 3 days at 4 days old. C and E show the proportion of phloem poles with or without gaps; B and D show mean±s.e.m. *P<0.05; **P<0.01; ***P<0.001 [Student's *t*-test (B,D) or Fisher's exact test (C,E)].

First, we examined whether, or to what degree, the *BRX* or *OPS* genes act through brassinosteroid signaling components. Similar to the hypocotyl, it has been suggested that threshold BR activity limits auxin activity in the root, because BR application rescues systemically reduced auxin response in *brx* root meristems (Gujas et al., 2012; Mouchel et al., 2006). Consistent with this, BR treatment also alleviated reduced auxin response in *ops* meristems (Rodriguez-Villalon et al., 2014) as indicated by the inverse auxin signaling reporter DII-VENUS (Santuari et al., 2011), whereas wild-type root meristems showed little detectable change (Fig. 1A). However, compared with *brx*, the effect of BR treatment in *ops* was less pronounced and an impact on root growth hardly detectable (Fig. 1B). To test whether BR treatment also affected *brx* or *ops* protophloem differentiation, we quantified cells that apparently failed to enter the sieve element differentiation program. These so-called ‘gap’ cells occur in the differentiation zone of the protophloem sieve element strands and are easily distinguished by their reduced propidium iodide cell wall staining (Scacchi et al., 2010; Truernit et al., 2012). Interestingly, BR-treated *ops*, but not *brx* mutants, showed statistically significant reduction in gap cell frequency (Fig. 1C). By contrast, no rescue was observed with respect to root growth or gap cell frequency in either genotype upon treatment with bikinin (Fig. 1D,E), a pharmacological GSK3 (and BIN2) inhibitor (De Rybel et al., 2009). Thus, BR pathway activation could partially rescue distinct aspects of the *brx* or *ops* phenotypes. Consistent with the notion that the observed effects reflect parallel action of BR signaling and *BRX*, combination of a loss-of-function allele in the main BR receptor, BRI1, with a *brx* null allele led to an additive root phenotype in homozygous *brx bri1* double mutants (Fig. S1A). Matching this observation, inspection by confocal microscopy also revealed an even more severe root meristem phenotype than in either single mutant (Fig. S1B). This included development of the protophloem, which displayed hardly any properly differentiating sieve elements. This phenotype was so severe that gap quantification became practically unfeasible. Moreover, in many *brx bri1* roots (∼40%) only a single protophloem strand could be detected.

### *bri1 brl1 brl3* triple mutants display protophloem differentiation defects

**Fig. 2.**
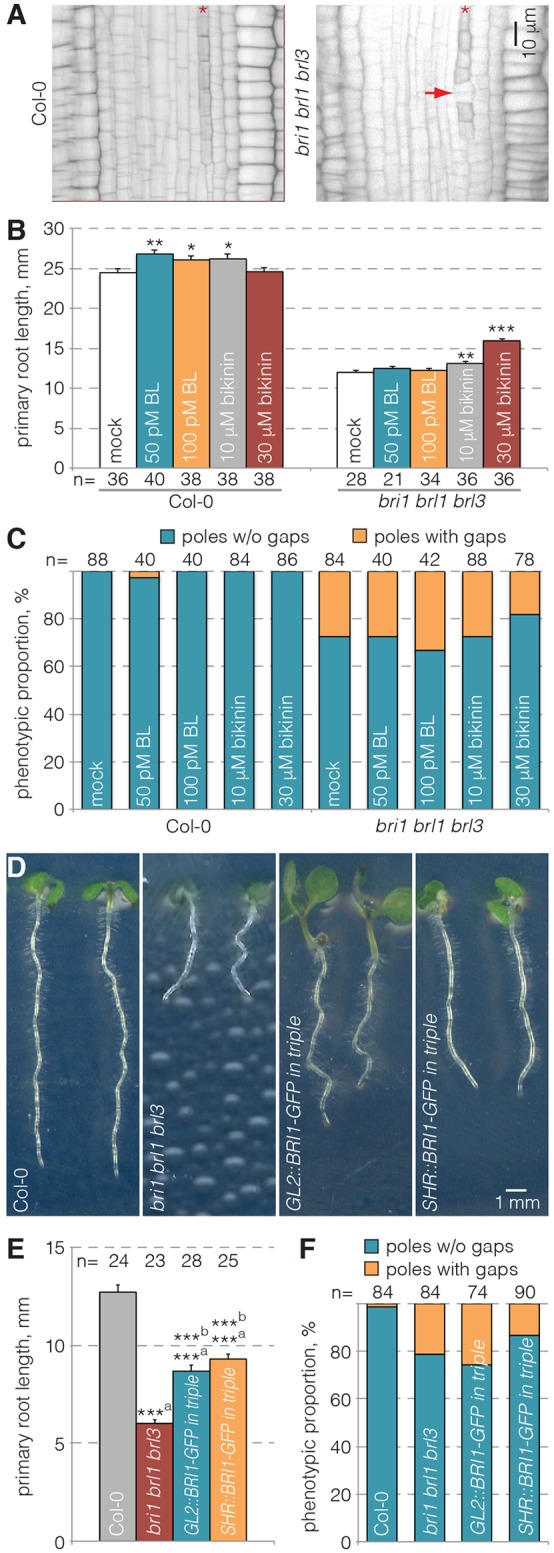
**Quantification of protophloem differentiation defects in brassinosteroid receptor mutants.** (A) Protophloem differentiation in Col-0 wild type or *bri1 brl1 brl3* brassinosteroid receptor (‘triple’) mutants (confocal microscopy, inverted gray scale). Asterisks indicate protophloem sieve element strands, arrowhead points out gap cells in the triple mutant. (B,C) Quantification of root growth (B) and gap frequency (C) in seedlings transferred onto indicated media for 3 days at 4 days old. (D-F) Macroscopic phenotype (D), root growth quantification (E) and gap frequency (F) of 5-day-old seedlings. C and F show the proportion of phloem poles with or without gaps; B and E show mean±s.e.m. *P<0.05; **P<0.01; ***P<0.001; ^a^versus Col-0; ^b^versus *bri1 brl1 brl3* [Student's *t*-test (B,E) or Fisher's exact test (C,F)].

To investigate further a possible involvement of the BR signaling pathway in protophloem differentiation, we next monitored the phenotype of *bri1* mutants in more detail. However, although *bri1* mutants displayed strongly reduced root growth, no gap cells were observed (*n*=44), consistent with previous reports (Anne et al., 2015). This could mean that phloem phenotypes were masked by redundancy or feedback systems. To avoid such ambiguous scenarios, we investigated *bri1 brl1 brl3* triple mutants (referred to hereafter as ‘triple mutant’), in which BR perception is completely shut down and therefore phenotypes are uncoupled from regulatory feedbacks on BR signal transduction or biosynthesis (Cano-Delgado et al., 2004). These experiments revealed that gap cells do indeed occur in *bri1 brl1 brl3* triple mutants (Fig. 2A), although at considerably lower frequency than in *brx* or *ops*. By contrast, *brl1 brl3* double mutant protophloem were wild type in appearance (*n*=26), corroborating the unequally redundant action of *BRL1* and *BRL3* (Briggs et al., 2006), as well as the nevertheless dominant role of *BRI1* in protophloem development. As expected, neither triple mutant protophloem defects nor overall root growth could be rescued by BR application. However, root growth, but not protophloem differentiation, was partially rescued by bikinin treatment (Fig. 2B,C).

Systemic propagation of local BR signaling via unknown pathways has been suggested as an explanation for non-cell-autonomous rescue of BR receptor mutants by epidermis-specific re-addition of *BRI1* activity (Hacham et al., 2011; Savaldi-Goldstein et al., 2007). With respect to the root, opposing, action site-dependent effects of BR signaling on root meristem size have been reported (Hacham et al., 2011; Vragović et al., 2015). Here, we analyzed whether protophloem differentiation responds to proposed non-cell-autonomous cues by investigating triple mutants that expressed functional *BRI-GFP* fusions under control of either the *GLABRA 2* (*GL2*) or the *SHORT ROOT* (*SHR*) promoter (hereafter *GL2::BRI1-GFP^triple^* and *SHR::BRI1-GFP^triple^*, respectively) (Vragović et al., 2015) (Fig. 2D). Whereas *GL2* confers expression in epidermal non-root-hair cell files, *SHR* drives expression throughout the stele. In both cases, the triple mutant short root phenotype was partially rescued (Fig. 2E), but protophloem differentiation defects were not (Fig. 2F). Although this was expected for *GL2::BRI1-GFP^triple^* plants, it was surprising for the *SHR::BRI1-GFP^triple^* plants. However, we found that *SHR::BRI1-GFP* was not expressed in the phloem poles (Fig. S1C), unlike *BRI1-GFP* expressed under control of its native promoter (Fig. S1D). Thus, the triple mutant protophloem defects could not be rescued by re-addition of *BRI1-GFP* to immediately neighboring cell files, arguing for a possibly cell-autonomous contribution of BR signaling to protophloem development.

### The small root meristem of *bri1 brl1 brl3* triple mutants can be entirely explained by reduced cell elongation

**Fig. 3.**
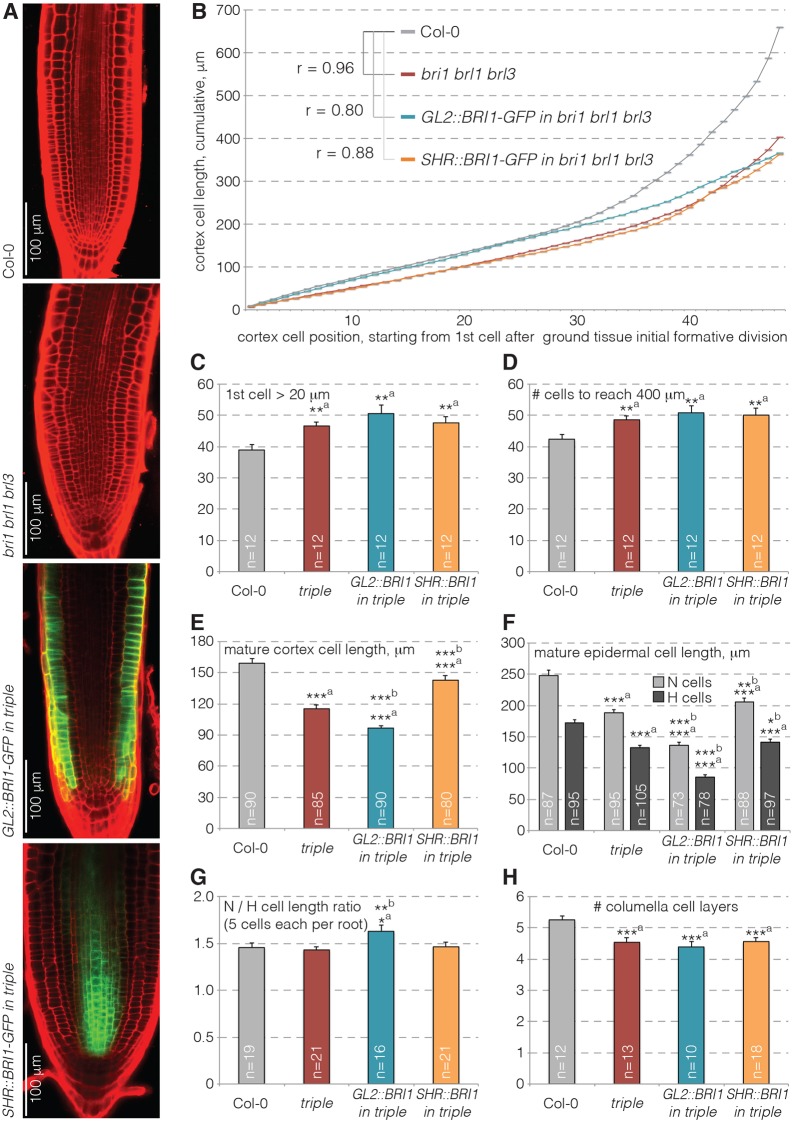
**Quantification of longitudinal meristem phenotypes in 5-day-old brassinosteroid receptor mutants.** (A) Confocal microscopy images of representative propidium iodide-stained (red) root meristems for the indicated genotypes. Note overlay with fluorescent GFP signal (green) in the partially complemented lines (same exposure settings). (B) Cumulative cell lengths along cortex cell files, averaged per position for six roots. r=Pearson correlation coefficient for curve comparison to Col-0. (C-H) Quantification of the position of first cortex cell to elongate beyond 20 µm (C), the number of cells along cortex cell files to reach 400 µm (D), mature cortex cell length (E), mature epidermal cell length for root hair cells (‘H’) and non-root hair cells (‘N’) (F), length ratio between epidermal H and N cells (G) and number of columella cell layers (H) for the genotypes shown in A. C-H show mean±s.e.m. *P<0.05; **P<0.01; ***P<0.001; ^a^versus Col-0; ^b^versus *bri1 brl1 brl3* (Student's *t*-test).

The absence of protophloem defects in *bri1* single mutants not only suggested BR receptor redundancy in protophloem development, but also that impaired protophloem differentiation is not the primary cause for the short root of *bri1* or the triple mutant. In part, this phenotype has been attributed to reduced meristem size (Fig. S1E), as indicated by the number of meristematic cells along root cell files (Gonzalez-Garcia et al., 2011; Hacham et al., 2011; Vragović et al., 2015). However, given the overall morphological irregularities in the triple mutant or its *GL2::BRI1-GFP^triple^* and *SHR::BRI1-GFP^triple^* derivatives (Fig. 3A), assigning the border between cell proliferation and elongation could become somewhat subjective. Therefore, we measured the progression of cell expansion along meristematic cortex files instead. Plots of cumulative cell length along the cortex files indicated that cell elongation is reduced in the triple mutant at all positions. However, the corresponding wild-type and triple mutant curves were highly correlated (Pearson's r=0.96) (Fig. 3B), indicating that the small *bri1 brl1 brl3* meristem can be entirely explained by reduced cell elongation, similar to the *dwarf4* BR biosynthetic mutant (Chaiwanon and Wang, 2015). In wild type, cell divisions were no longer observed once cells elongated above 20 µm. In the triple mutant or its derivatives, 20 µm length was reached about ten cells later than in wild type (Fig. 3C), and cell files contained about eight more cells at 400 µm (Fig. 3D). However, because cell elongation was generally reduced, cell divisions in the mutant lines apparently ceased earlier, before the 20 µm mark. Moreover, correlation between *GL2::BRI1-GFP^triple^* or *SHR::BRI1-GFP^triple^* and wild type was still high (r=0.80 and 0.88, respectively), and even higher when compared with *bri1 brl1 brl3* (r=0.84 and 0.89, respectively), consistent with no statistically significant difference between *GL2::BRI1-GFP^triple^* and *SHR::BRI1-GFP^triple^* and the triple mutant in other parameters (Fig. 3C,D).

**Fig. 4.**
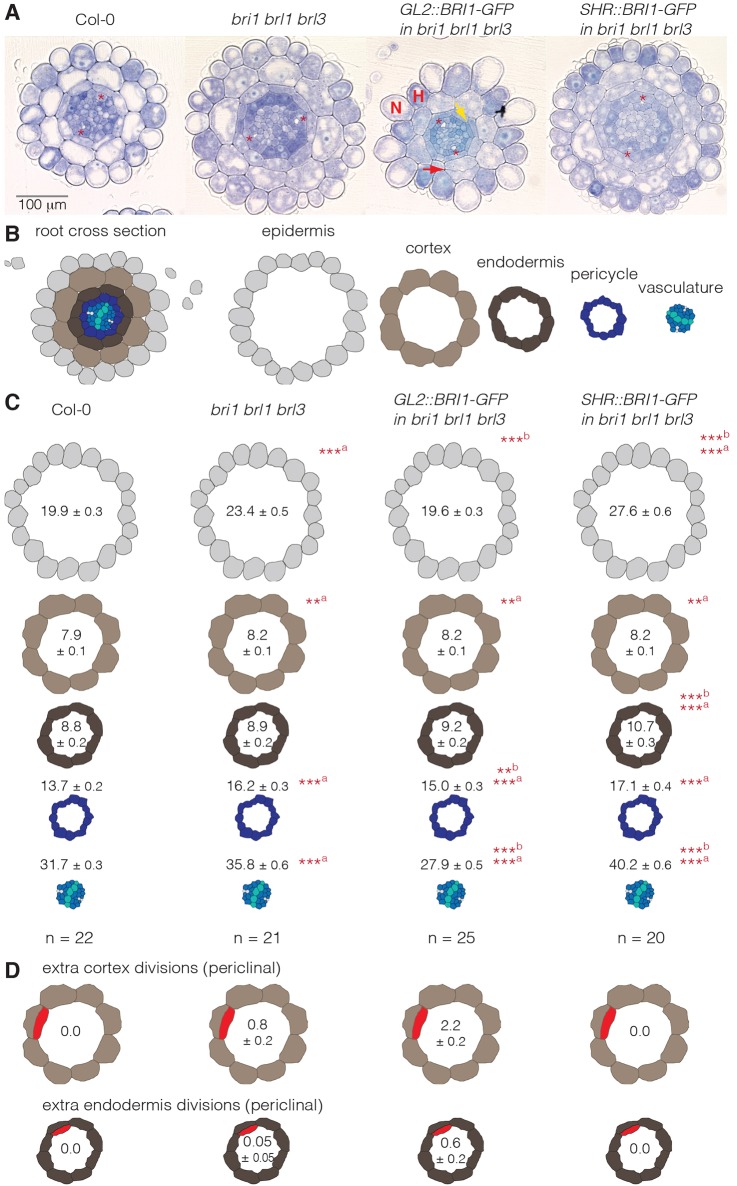
**Quantification of radial meristem phenotypes in 5-day-old brassinosteroid receptor mutants.** (A) Toluidine Blue-stained root sections, taken at the position where protophloem poles (asterisks) have already differentiated, but protoxylem has not. Epidermal H and N cells are indicated for *GL2::BRI1-GFP^triple^*. Red arrow points out an extra cortex division, yellow arrow an extra endodermis division. (B) Schematics of the different root tissue layers in a cross-section of a Col-0 wild-type plant. (C) Quantification (mean±s.e.m.) of cell number in the different tissues. (D) Quantification (mean±s.e.m.) of extra cortex or endodermis divisions. **P<0.01; ***P<0.001; ^a^versus Col-0; ^b^versus *bri1 brl1 brl3* (Student's *t*-test).

Interestingly, statistically significant, but small partial rescue of reduced mature cell size, as exemplified by cortex (Fig. 3E) and epidermis (Fig. 3F) cells, was observed in *SHR::BRI1-GFP^triple^* plants (Fig. 3E,F). By contrast, *GL2::BRI1-GFP^triple^* plants displayed even further reduced mature cell length, as previously reported (Fridman et al., 2014). However, reduced root growth was still weakly complemented by *GL2::BRI1-GFP*, even after a longer growth period (Fig. S1F). Notably, however, the phenotype of these plants was difficult to assess because their roots twisted, which could result from the fact that *GL2::BRI1-GFP* is only expressed in non-root-hair cell files. The increased ratio of non-hair cell length to hair cell length might reflect this (Fig. 3G). Transverse sections showed that *BRI1-GFP*-expressing epidermal cells also expanded considerably more radially than their immediate hair cell neighbors (Fig. 4A), confirming earlier reports (Fridman et al., 2014), which is likely to create mechanical strain between them.

### Cell division rate in the radial dimension reacts to cell-autonomous as well as non-cell-autonomous cues

Consistent with previous reports that BR signaling promotes columella stem cell divisions (Chaiwanon and Wang, 2015; Gonzalez-Garcia et al., 2011), *bri1 brl1 brl3* mutants displayed slightly reduced columella cell layers, which was not rescued in the derivative lines (Fig. 3H). By contrast, the number of formative cell divisions was markedly increased in the radial dimension across all cell layers except the endodermis, giving rise to additional cell files (Fig. 4A-C). Moreover, extra periclinal cortex and endodermis divisions were observed occasionally in the triple mutant, but not in wild type (Fig. 4D). This phenotype was already observed in a *bri1* single mutant, although it was not observed as often as in the triple mutant (Fig. S2A). Interestingly, our observations fit with previously reported increased cyclin B1 activity in the tip of *bri1* mutant root meristems (Gonzalez-Garcia et al., 2011) (with the caveat that this is not necessarily indicative of more cell divisions). Together with the reduced cell expansion described above, this observation would also explain the overall strongly reduced root growth of *bri1 brl1 brl3* mutants, because accommodation of supernumerary formative divisions could presumably slow down overall root elongation.

Surprisingly, the *GL2::BRI1-GFP* and *SHR::BRI1-GFP* transgenes modulated the radial increase in cell files in a unique, opposite fashion. Whereas *GL2::BRI1-GFP^triple^* plants basically complemented, and sometimes even over-compensated the excessive radial cell proliferation, the triple mutant phenotype was further enhanced in *SHR::BRI1-GFP^triple^* plants (Fig. 4A-C). Thus, the cell division pattern appeared to accommodate local variation in BR signaling in a partly cell-autonomous, partly non-cell-autonomous fashion. The observations also suggest that overall root growth was partially rescued in both *GL2::BRI1-GFP^triple^* and *SHR::BRI1-GFP^triple^* plants for different reasons. Whereas the excess formative cell divisions, but not the reduced cell elongation, were partially compensated in *GL2::BRI1-GFP^triple^* plants, the inverse applied to *SHR::BRI1-GFP^triple^* plants.

### BRI1 activity in the developing protophloem is sufficient to rescue all observed major root phenotypes of *bri1 brl1 brl3* triple mutants

**Fig. 5.**
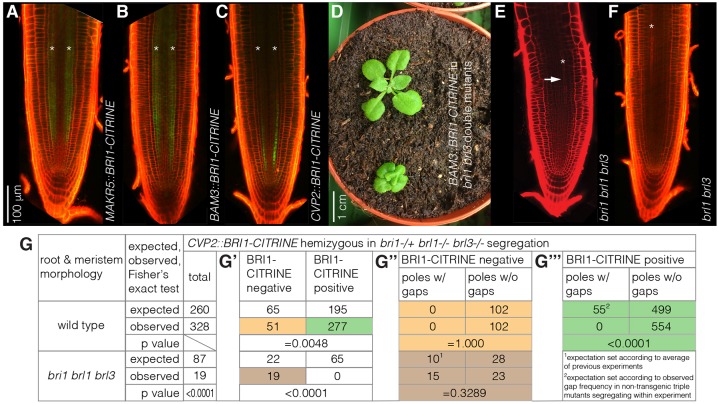
**Rescue of *bri1 brl1 brl3* triple mutant root phenotypes by phloem-specific *BRI1* expression.** (A-C) Confocal microscopy images of representative root meristems for indicated transgenes, showing BRI1-CITRINE expression (green fluorescence) in the developing protophloem. Red, propidium iodide. (D) Rosette phenotypes of *brl1 brl3* double mutants that express a *BAM3::BRI1-CITRINE* transgene. (E,F) Root meristems of *bri1 brl1 brl3* triple mutants (E) and *brl1 brl3* double mutants (F) segregating from a *bri1*^+/−^ *brl1*^−/−^ *brl3*^−/−^ mother plant that carried a hemizygous *CVP2::BRI1-CITRINE* transgene. Note the autofluorescence background in A-C as exemplified by (non-transgenic) F (these images were taken with the same confocal settings). (G) Phenotypic analysis of the segregating progeny of a *bri1*^+/−^ *brl1*^−/−^ *brl3*^−/−^ mother plant that carried a hemizygous single insertion of a *CVP2::BRI1-CITRINE* transgene. Overall root growth, meristem appearance and protophloem gaps were scored for an entire sample of 347 seedlings, indicating rescue of the *bri1 brl1 brl3* short root phenotype (G,G′) as well as the protophloem phenotype (G″,G‴) by the transgene. Asterisks indicate protophloem sieve element strands. Arrow in E indicates gap cells. Colors in G″ and G‴ refer to the subgroups indicated in G′.

Interestingly, in the F1 derived from crosses between *GL2::BRI1-GFP^triple^* and *SHR::BRI1-GFP^triple^* plants, the formative cell division phenotypes of the two parental lines largely compensated each other (Fig. S2B). At the same time, both mature cortex cell length and overall root growth were restored to nearly wild-type levels (Fig. S2,D). However, gap cells still occurred in these plants and, although their frequency was reduced, this was only borderline statistically significant compared with the parental lines (Fig. S2E). To evaluate directly the contribution of BR signaling to protophloem development, we therefore created transgenic lines in which we expressed a BRI1-CITRINE fusion protein under control of different, increasingly protophloem-specific promoters. The *MEMBRANE-ASSOCIATED KINASE REGULATOR 5* (*MAKR5*) promoter confers expression in the phloem poles in the early meristem, and throughout the root vasculature later on (Kang and Hardtke, 2016) (Fig. 5A). By contrast, the *BARELY ANY MERISTEM 3* (*BAM3*) promoter drives expression at the phloem pole from early on, with its strongest activity in the developing sieve elements and possibly some weak activity in companion cells (Depuydt et al., 2013; Rodriguez-Villalon et al., 2014) (Fig. 5B). Finally, the *COTYLEDON VASCULAR PATTERN 2* (*CVP2*) promoter is very specific for the developing sieve element cell file (Rodriguez-Villalon et al., 2015) (Fig. 5C). Analysis of lines that carried the *BAM3::BRI1-CITRINE* transgene were complicated by the observation that it triggered an apparently dosage-dependent shoot phenotype (Fig. 5D). However, when *MAKR5::BRI1-CITRINE* or *CVP2::BRI1-CITRINE* transgenes were introduced into *brl1 brl3* double mutant plants that were heterozygous for the *bri1* mutation, surprisingly only two distinct phenotypic classes were observed in the next generation, in multiple, independent transgenic lines (Fig. S2F). The first class comprised short-rooted individuals corresponding to *bri1 brl1 brl3* triple mutants (Fig. 5E), and the second class comprised individuals with fully developed roots with a wild-type appearance that were also similar to *brl1 brl3* double mutant roots (Fig. 5F). The two phenotypic classes appeared in ratios of approximately 1:15, suggesting that both constructs fully rescued the short root phenotype of the triple mutant. More detailed analyses confirmed this notion. For instance, in the progeny of a *bri1*^−/+^ *brl1*^−/−^ *brl3*^−/−^ plant that carried a single *CVP2::BRI1-CITRINE* T-DNA insertion, 11 short root individuals and 165 long root individuals were observed. This segregation ratio was significantly different from 1/4 (*P*<0.0001; Fisher's exact test), but not from 1/16. Genotyping confirmed that all the 11 short root individuals were *bri1 brl1 brl3* triple mutants and did not carry the transgene, consistent with the absence of CITRINE signal in their roots. Moreover, the meristems of seedlings with long roots that expressed the *CVP2::BRI1-CITRINE* transgene were essentially indistinguishable from the meristems of seedlings with long roots that were not transgenic (i.e. *brl1 brl3* double mutants, or such double mutants heterozygous for *bri1*). This included 25% of complemented triple mutants, as we did not observe aborted seeds or arrested, non-germinating seedlings. In summary, in all individuals that expressed the transgenes, we observed long roots with a growth rate equal to wild type, restored cell elongation, and absence of protophloem gaps. To confirm this finding with higher statistical confidence, we analyzed an entire progeny of 347 seedlings segregating from another, independent *bri1*^−/+^ *brl1*^−/−^ *brl3*^−/−^ plant that carried a single hemizygous *CVP2::BRI1-CITRINE* T-DNA insertion in confocal microscopy. Again, not a single protophloem gap was observed in meristems with wild-type appearance (Fig. 5G), demonstrating that the transgene complemented not only the reduced root growth and altered meristem morphology of *bri1 brl1 brl3* triple mutants, but also their protophloem differentiation defect. However, we could not conclusively determine whether the transgenes also rescued the increase in formative cell divisions, because the presence of the transgenic *BRI1* copy prevented conclusive genotyping of the (intron-less) endogenous locus except to confirm presence or absence of the mutant allele. We also could not recover triple mutants that were homozygous for the transgenes, because the shoot phenotype was not fully rescued. Therefore, with the possible exception of restricting formative cell divisions, we have shown that protophloem-specific BR signaling is sufficient to mediate the major effects of systemic BR action throughout the root meristem.

## DISCUSSION

In this study, we evaluated whether BR signaling has a role in root protophloem development as recently proposed by the finding that the OPS protein can interact with, and thereby inhibit, BIN2 (Anne et al., 2015). We did indeed observe a substantial contribution of BR signaling to the progression of sieve element differentiation in BR receptor triple mutants, which appeared to be moderate, however, when compared with *brx* or *ops* mutants. Our findings suggest that OPS action cannot be entirely mediated through BIN2 and its downstream targets. In part, our results are incongruent with previous studies. For example, whereas it has been reported that bikinin treatment, but not BR treatment, could rescue *ops* gap cells (Anne et al., 2015), we observed the inverse. Such divergent outcomes could be explained by different assay or sampling conditions, or by small sample sizes that can lead to statistically significant yet not reproducible outcomes in individual replicate experiments. Unfortunately, this limitation is sometimes difficult to overcome because of the technical challenge and the major time investment associated with microscopic investigation of the protophloem. However, the notion emerging from our results that any OPS effects on BIN2 activity might not be mediated by the canonical downstream factors of BR signaling is also supported by the relatively small, statistically borderline significant effect of dominant *bzr1-D* or *bes1-D* mutation on *ops* root phenotypes (Anne et al., 2015), especially when compared with the substantial *bzr1-D* or *bes1-D* rescue of *bri1* root growth (Chaiwanon and Wang, 2015). The finding that *bzr1-D* or *bes1-D* mutations have no effect on *ops* cotyledon vasculature phenotypes, and that OPS gain-of-function suppresses *bri1* and *bin2* dwarfism (Anne et al., 2015) supports this idea. Moreover, given that the link between BIN2 and OPS is so far exclusively based on observations in OPS gain-of-function scenarios, it still appears possible that BIN2 inhibition by OPS might represent a neomorphic or context-specific scenario. Indeed, our finding that BR signaling contributes to protophloem differentiation, but that GSK3 or BIN2 inhibition is not sufficient to substitute for loss of BR signaling in this context, supports this notion and is also consistent with normal protophloem development in *bin2* gain-of-function mutants (Anne et al., 2015). Therefore, it still appears possible that BR perception contributes to protophloem differentiation through a GSK3/BIN2-independent route, or through one of the few GSK3 proteins that do not respond to bikinin. Alternatively, the impact of BR signaling on sieve element differentiation might be indirect. For instance, it is conceivable that the loss of BR receptors might increase the accessible pool of their co-receptors, which could then result in stoichiometric shifts towards other receptor signaling systems, such as the CLE45-BAM3 pathway, which suppresses protophloem differentiation (Depuydt et al., 2013). Our observation that the root phenotypes of *brx* and *bri1* mutants are additive would be consistent with a parallel, possibly indirect, action of BR signaling in protophloem development.

Our observations suggest that in the root BR signaling is primarily required for proper cell elongation and to restrict the extent of formative divisions. Our finding that *bri1 brl1 brl3* triple mutants produce more root cell files also matches with a previously reported increase of cyclin B1 activity in the tip of *bri1* mutant root meristems (Gonzalez-Garcia et al., 2011), which already display this phenotype. Our data also indicate that meristem size in *bri1 brl1 brl3* triple mutants can be entirely explained by reduced cell elongation. This might seem surprising given previous reports of altered meristematic cell number in brassinosteroid mutants (Gonzalez-Garcia et al., 2011; Hacham et al., 2011; Chaiwanon and Wang, 2015; Vragović et al., 2015). However, the reported changes were typically rather small and not always congruent between studies. This could reflect different assay conditions and/or genetic materials, but also different quantification strategies. For instance, some authors quantified meristematic cortex cells (Hacham et al., 2011; Chaiwanon and Wang, 2015), whereas others scored epidermal cell files (Gonzalez-Garcia et al., 2011). The latter approach could be misleading if quantification is not consistently done in trichoblast or atrichoblast cell files only, because cell division and elongation rate are different in the two. In our study, we found that reduced cell elongation can by itself explain the small meristem size of *bri1 brl1 brl3* triple mutants. However, it apparently cannot explain the relatively higher overall root growth reduction (i.e. ∼50% reduction in root length compared with ∼25% reduction in mature cell length). One explanation for this growth reduction would be an influence of BR signaling on the balance between cell proliferation and differentiation across cell layers, as previously proposed (Vragović et al., 2015). Alternatively, *bri1 brl1 brl3* roots might simply be growing slowly, because the supernumerary formative cell divisions have to be accommodated in space and time. In support of the latter idea, a similar short root phenotype can be created artificially by transgenic induction of formative cell divisions (De Rybel et al., 2013).

Perhaps our most surprising result was the observation that BRI1 activity that was restricted to the developing protophloem could rescue all major aspects of the *bri1 brl1 brl3* triple mutant root phenotype. Such non-cell-autonomous effects of BR signaling have been described extensively before (Fridman et al., 2014; Hacham et al., 2011; Savaldi-Goldstein et al., 2007; Vragović et al., 2015); however, their penetrance was typically found to be partial. Another example of non-cell-autonomous BR effects is our finding that increased stele cell divisions were suppressed in *GL2::BRI1-GFP^triple^* plants, whereas epidermal cell divisions were increased in *SHR::BRI1-GFP^triple^* plants, and combination of the two transgenes led to their mutual compensation and thus an intermediate phenotype. This points to flexible non-cell-autonomous accommodations of local growth effects, which could result from physiological consequences of site-specific BR signaling action as proposed by transcriptomic analyses (Chaiwanon and Wang, 2015; Vragović et al., 2015). Alternatively, they could reflect reactions to mechanical stress. Irrespective of the mechanism, protophloem-specific BR perception appears to be sufficient to systemically convey the major effects of BR action in the root meristem context, once more underlining the essential function of the protophloem for overall root system growth and organization (Depuydt et al., 2013; Rodriguez-Villalon et al., 2015).

## MATERIALS AND METHODS

### Plant materials and phenotyping

The mutants and transgenic lines used in this study were all in Col-0 background and most have been described before (Geldner et al., 2007; Rodriguez-Villalon et al., 2014; Scacchi et al., 2010; Vragović et al., 2015). Plant tissue culture, (confocal) microscopy, histology and physiological assays were performed according to standard procedures (Rodriguez-Villalon et al., 2014, 2015). Brassinolide was purchased from Sigma, bikinin from Wako Chemicals.

### Genotyping and transgenic lines

Genotyping of *brx* alleles was performed as described (Mouchel et al., 2004; Rodriguez-Villalon et al., 2014). Genotyping of other loci by analysis of PCR-amplified genomic DNA fragments was performed as follows. To detect the *bri1-116* allele, a 552 bp genomic DNA fragment was amplified using oligonucleotides 5′-AAGGAGAGATCCCTCAGGAG-3′ and 5′-TGTCCAGAAACATCATCGAAC-3′. Restriction digest with *Pme*I indicated presence of the *bri1-116* allele if amplicons could not be cut into 314 bp and 238 bp fragments. To detect the *BRL1* alleles, oligonucleotide 5′-CTGTAAAGCGCCATGACTAGC-3′ was combined with either 5′-ATATGGATGTTGCCGAATCTG-3′ (to detect the *BRL1* wild-type allele) or 5′-ATTTTGCCGATTTCGGAAC-3′ (to detect the *brl1* T-DNA insertion). To detect the *BRL3* alleles, oligonucleotide 5′-TTTATCGAACACTTTGTGGGC-3′ was combined with either 5′-CCAGTGAACTCGTTTGAGCTC-3′ (to detect the *BRL3* wild-type allele) or 5′-ATTTTGCCGATTTCGGAAC-3′ (to detect the *brl3* T-DNA insertion). To detect the *bri1-1* allele, a 224 bp genomic DNA fragment was amplified using oligonucleotides 5′-GCTAACAACACCAATTGGAAG-3′ and 5′-CTAACATGAATCAGTTCTTGATAT-3′. Restriction digest with *Eco*RV indicated presence of the *bri1-1* allele if amplicons were cut into 201 bp and 23 bp fragments. PCR annealing temperature was 56°C, with an extension time of 1 min. The *MAKR5::BRI1-CITRINE*, *BAM3::BRI1-CITRINE* and *CVP2::BRI-CITRINE* constructs were created by replacing the respective coding sequences in described vectors (Kang and Hardtke, 2016; Rodriguez-Villalon et al., 2014, 2015) with the BRI1 coding sequence. The constructs were then transformed into homozygous *brl1 brl3* double mutants that were heterozygous for the *bri1-116* allele, and similar *brl1*^−/−^ *brl3*^−/−^ *bri1*^+/−^ plants that carried the transgenes were selected by genotyping in the T2 generation. Phenotypic analyses were performed in the subsequent T3 generation.

## References

[DEV145623C1] Anne P., Azzopardi M., Gissot L., Beaubiat S., Hématy K. and Palauqui J.-C. (2015). OCTOPUS negatively regulates BIN2 to control phloem differentiation in Arabidopsis thaliana. *Curr. Biol.* 25, 2584-2590. 10.1016/j.cub.2015.08.033 26387715

[DEV145623C2] Briggs G. C., Osmont K. S., Shindo C., Sibout R. and Hardtke C. S. (2006). Unequal genetic redundancies in Arabidopsis--a neglected phenomenon? *Trends Plant Sci.* 11, 492-498. 10.1016/j.tplants.2006.08.005 16949326

[DEV145623C3] Cano-Delgado A., Yin Y., Yu C., Vafeados D., Mora-Garcia S., Cheng J.-C., Nam K. H., Li J. and Chory J. (2004). BRL1 and BRL3 are novel brassinosteroid receptors that function in vascular differentiation in Arabidopsis. *Development* 131, 5341-5351. 10.1242/dev.01403 15486337

[DEV145623C4] Chaiwanon J. and Wang Z.-Y. (2015). Spatiotemporal brassinosteroid signaling and antagonism with auxin pattern stem cell dynamics in Arabidopsis roots. *Curr. Biol.* 25, 1031-1042. 10.1016/j.cub.2015.02.046 25866388 PMC4415608

[DEV145623C5] Cho H., Ryu H., Rho S., Hill K., Smith S., Audenaert D., Park J., Han S., Beeckman T., Bennett M. J. et al. (2014). A secreted peptide acts on BIN2-mediated phosphorylation of ARFs to potentiate auxin response during lateral root development. *Nat. Cell Biol.* 16, 66-76. 10.1038/ncb2893 24362628

[DEV145623C6] De Rybel B., Audenaert D., Vert G., Rozhon W., Mayerhofer J., Peelman F., Coutuer S., Denayer T., Jansen L., Nguyen L. et al. (2009). Chemical inhibition of a subset of Arabidopsis thaliana GSK3-like kinases activates brassinosteroid signaling. *Chem. Biol.* 16, 594-604. 10.1016/j.chembiol.2009.04.008 19549598 PMC4854203

[DEV145623C7] De Rybel B., Möller B., Yoshida S., Grabowicz I., Barbier de Reuille P., Boeren S., Smith R. S., Borst J. W. and Weijers D. (2013). A bHLH complex controls embryonic vascular tissue establishment and indeterminate growth in Arabidopsis. *Dev. Cell* 24, 426-437. 10.1016/j.devcel.2012.12.013 23415953

[DEV145623C8] Depuydt S., Rodriguez-Villalon A., Santuari L., Wyser-Rmili C., Ragni L. and Hardtke C. S. (2013). Suppression of Arabidopsis protophloem differentiation and root meristem growth by CLE45 requires the receptor-like kinase BAM3. *Proc. Natl. Acad. Sci. USA* 110, 7074-7079. 10.1073/pnas.1222314110 23569225 PMC3637694

[DEV145623C9] Fridman Y., Elkouby L., Holland N., Vragović K., Elbaum R. and Savaldi-Goldstein S. (2014). Root growth is modulated by differential hormonal sensitivity in neighboring cells. *Genes Dev.* 28, 912-920. 10.1101/gad.239335.114 24736847 PMC4003282

[DEV145623C10] Geldner N., Hyman D. L., Wang X., Schumacher K. and Chory J. (2007). Endosomal signaling of plant steroid receptor kinase BRI1. *Genes Dev.* 21, 1598-1602. 10.1101/gad.1561307 17578906 PMC1899468

[DEV145623C11] Gonzalez-Garcia M.-P., Vilarrasa-Blasi J., Zhiponova M., Divol F., Mora-Garcia S., Russinova E. and Cano-Delgado A. I. (2011). Brassinosteroids control meristem size by promoting cell cycle progression in Arabidopsis roots. *Development* 138, 849-859. 10.1242/dev.057331 21270057

[DEV145623C12] Grove M. D., Spencer G. F., Rohwedder W. K., Mandava N., Worley J. F., Warthen J. D., Steffens G. L., Flippen-Anderson J. L. and Cook J. C. (1979). Brassinolide, a Plant Growth-Promoting Steroid Isolated from Brassica Napus Pollen. *Nature* 281, 216-217. 10.1038/281216a0

[DEV145623C13] Gujas B., Alonso-Blanco C. and Hardtke C. S. (2012). Natural Arabidopsis brx loss-of-function alleles confer root adaptation to acidic soil. *Curr. Biol.* 22, 1962-1968. 10.1016/j.cub.2012.08.026 23041192

[DEV145623C14] Hacham Y., Holland N., Butterfield C., Ubeda-Tomas S., Bennett M. J., Chory J. and Savaldi-Goldstein S. (2011). Brassinosteroid perception in the epidermis controls root meristem size. *Development* 138, 839-848. 10.1242/dev.061804 21270053 PMC3035089

[DEV145623C15] Kang Y. H. and Hardtke C. S. (2016). Arabidopsis MAKR5 is a positive effector of BAM3-dependent CLE45 signaling. *EMBO Rep.* 17, 1145-1154. 10.15252/embr.201642450 27354416 PMC4967951

[DEV145623C16] Kondo Y., Ito T., Nakagami H., Hirakawa Y., Saito M., Tamaki T., Shirasu K. and Fukuda H. (2014). Plant GSK3 proteins regulate xylem cell differentiation downstream of TDIF-TDR signalling. *Nat. Commun.* 5, 3504 10.1038/ncomms4504 24662460

[DEV145623C17] Li J. and Chory J. (1997). A putative leucine-rich repeat receptor kinase involved in brassinosteroid signal transduction. *Cell* 90, 929-938. 10.1016/S0092-8674(00)80357-8 9298904

[DEV145623C18] Li J. and Nam K. H. (2002). Regulation of brassinosteroid signaling by a GSK3/SHAGGY-like kinase. *Science* 295, 1299-1301. 10.1126/science.1065769 11847343

[DEV145623C19] Mouchel C. F., Briggs G. C. and Hardtke C. S. (2004). Natural genetic variation in Arabidopsis identifies BREVIS RADIX, a novel regulator of cell proliferation and elongation in the root. *Genes Dev.* 18, 700-714. 10.1101/gad.1187704 15031265 PMC387244

[DEV145623C20] Mouchel C. F., Osmont K. S. and Hardtke C. S. (2006). BRX mediates feedback between brassinosteroid levels and auxin signalling in root growth. *Nature* 443, 458-461. 10.1038/nature05130 17006513

[DEV145623C21] Nemhauser J. L., Mockler T. C. and Chory J. (2004). Interdependency of brassinosteroid and auxin signaling in Arabidopsis. *PLoS Biol.* 2, e258 10.1371/journal.pbio.0020258 15328536 PMC509407

[DEV145623C22] Ohnishi T., Szatmari A.-M., Watanabe B., Fujita S., Bancos S., Koncz C., Lafos M., Shibata K., Yokota T., Sakata K. et al. (2006). C-23 hydroxylation by Arabidopsis CYP90C1 and CYP90D1 reveals a novel shortcut in brassinosteroid biosynthesis. *Plant Cell* 18, 3275-3288. 10.1105/tpc.106.045443 17138693 PMC1693957

[DEV145623C23] Rodriguez-Villalon A., Gujas B., Kang Y. H., Breda A. S., Cattaneo P., Depuydt S. and Hardtke C. S. (2014). Molecular genetic framework for protophloem formation. *Proc. Natl. Acad. Sci. USA* 111, 11551-11556. 10.1073/pnas.1407337111 25049386 PMC4128119

[DEV145623C24] Rodriguez-Villalon A., Gujas B., van Wijk R., Munnik T. and Hardtke C. S. (2015). Primary root protophloem differentiation requires balanced phosphatidylinositol-4,5-biphosphate levels and systemically affects root branching. *Development* 142, 1437-1446. 10.1242/dev.118364 25813544

[DEV145623C25] Santuari L., Scacchi E., Rodriguez-Villalon A., Salinas P., Dohmann E. M. N., Brunoud G., Vernoux T., Smith R. S. and Hardtke C. S. (2011). Positional information by differential endocytosis splits auxin response to drive Arabidopsis root meristem growth. *Curr. Biol.* 21, 1918-1923. 10.1016/j.cub.2011.10.002 22079112

[DEV145623C26] Savaldi-Goldstein S., Peto C. and Chory J. (2007). The epidermis both drives and restricts plant shoot growth. *Nature* 446, 199-202. 10.1038/nature05618 17344852

[DEV145623C27] Scacchi E., Salinas P., Gujas B., Santuari L., Krogan N., Ragni L., Berleth T. and Hardtke C. S. (2010). Spatio-temporal sequence of cross-regulatory events in root meristem growth. *Proc. Natl. Acad. Sci. USA* 107, 22734-22739. 10.1073/pnas.1014716108 21149702 PMC3012524

[DEV145623C28] Truernit E., Bauby H., Belcram K., Barthelemy J. and Palauqui J.-C. (2012). OCTOPUS, a polarly localised membrane-associated protein, regulates phloem differentiation entry in Arabidopsis thaliana. *Development* 139, 1306-1315. 10.1242/dev.072629 22395740

[DEV145623C29] Vragović K., Sela A., Friedlander-Shani L., Fridman Y., Hacham Y., Holland N., Bartom E., Mockler T. C. and Savaldi-Goldstein S. (2015). Translatome analyses capture of opposing tissue-specific brassinosteroid signals orchestrating root meristem differentiation. *Proc. Natl. Acad. Sci. USA* 112, 923-928. 10.1073/pnas.1417947112 25561530 PMC4311806

[DEV145623C30] Yin Y., Wang Z.-Y., Mora-Garcia S., Li J., Yoshida S., Asami T. and Chory J. (2002). BES1 accumulates in the nucleus in response to brassinosteroids to regulate gene expression and promote stem elongation. *Cell* 109, 181-191. 10.1016/S0092-8674(02)00721-3 12007405

[DEV145623C31] Zhu J.-Y., Sae-Seaw J. and Wang Z.-Y. (2013). Brassinosteroid signalling. *Development* 140, 1615-1620. 10.1242/dev.060590 23533170 PMC3621480

